# On a Rare Cutaneous Metastasis from a Sacrococcygeal Chordoma

**DOI:** 10.1155/2017/5281239

**Published:** 2017-03-19

**Authors:** Alessandro D'Amuri, Matteo Brunelli, Federica Floccari, Francesco De Caro, Giuliana Crisman, Francesca Sanguedolce, Marcello Filotico

**Affiliations:** ^1^Anatomic Pathology Unit, Card. G. Panico Hospital, Tricase, Italy; ^2^Department of Diagnostics and Public Health, Anatomic Pathology, University and Hospital Trust, Verona, Italy; ^3^Clinic Pathology Unit, Sacro Cuore di Gesù Hospital, Gallipoli, Italy; ^4^Orthopaedics and Traumatology Unit, Sacro Cuore di Gesù Hospital, Gallipoli, Italy; ^5^Pathology Unit, Desenzano del Garda General Hospital, Brescia, Italy; ^6^Department of Pathology, University and Hospital Trust, Riuniti Hospital, Foggia, Italy

## Abstract

Chordomas are rare malignant tumors of notochordal origin and are rare locally aggressive ones with a metastatic potential. The skin rarely is seen as metastatic site. We describe a case of an adult woman with cutaneous metastasis of a primary sacral chordoma excised ten years before, which appeared as a painless cutaneous mass located in the dorsal region. Once removed, the surgical specimen was formalin fixed and in paraffin embedded. Sections were stained with haematoxylin-eosin, and histochemical and immunohistochemical investigations were performed. Histologically, the neoplasia was characterized by cords or single tumor cells with an abundant myxoid stroma, conspicuous pale vacuolated cytoplasm (the classic “*physaliphorous cells*”), and mild nuclear atypia. Mitotic activity was scanty. At immunohistochemistry, the tumor cells were diffusely positive for S-100 protein, pan-keratins, EMA, and vimentin. A diagnosis of cutaneous metastasis of chordoma was performed. This case illustrates a diagnostic challenge because of the unusual presentation of an already rare tumor.

## 1. Introduction

Chordomas are uncommon malignant tumors originating from remnants of the notochord [1], vestigial transient organ, which forms the initial model of the spine at early stages of embryonal development. They mainly develop in the sacrococcygeal (50%) and sphenooccipital regions (35%) as a result of the presence of notochordal remnants in the clivus and in sacrococcygeal area. The persistence of aberrant chordal tissue has been reported in literature [2, 3]. Chordomas grow fairly slowly but they can relapse and have an aggressive clinical course metastasizing to other organs (ranging from 3% to 48% of cases), for example, the lungs, lymph nodes, liver, bones, skin, and skeletal muscle, or to the tissues adjacent to the primary tumor by direct extension or by local recurrence after surgical excision [1]. Some cases of cutaneous metastasis diagnosed before the primary tumor have been described [4, 5].

Chordomas may affect people of a wide age range with a peak of incidence between the fourth and the seventh decade of life, with a male to female ratio of 2 : 1. They sometimes occur in childhood, with an aggressive behaviour and rapid growth [1, 3].

The most common clinical presentation is represented by headache, vision disturbance (skull chordomas), and pain or nerve pressing (neck, back, and tailbone chordomas); there is however, a straight correlation with tumor location [6].

Skin represents an unusual site of onset for chordoma metastasis [7], occurring by direct extension, local recurrence, and metastasis. Herein we report on a case of a 68-year-old Caucasian woman with a cutaneous metastasis of a sacrococcygeal chordoma, excised ten years before. Histopathological and immunohistochemical findings are deepened and discussed.

## 2. Case Report

A 68-year-old Caucasian woman presented with a history of few months of a painless subcutaneous mass located in her right dorsal region (upper back near scapular region). A CT scan evaluation revealed a solid subcutaneous lesion with well-defined margins, ovoid in shape, of 4 cm diameter. The patient referred to an history of a sacral chordoma ten years before and subsequently underwent a surgical excision and a radiation therapy to reduce the potential for clinical relapse due to the positivity of the margins.

Grossly the specimen consisted of a capsulated mass of gelatinous and elastic consistency, with a yellowish-brown appearance for the presence of cystic degeneration and hemorrhagic areas.

All specimens were fixed in 4% buffered formalin, routinely processed, and embedded in paraffin; 4-micrometer thick sections were stained routinely with haematoxylin and eosin and enzymatically histochemically stained with Alcian-PAS. In addition, sections were stained immunohistochemically by the labeled avidin-biotin-peroxidase complex technique for vimentin, CEA, S100 protein, cytokeratin AE1/AE3, and smooth muscle actin. All the specimens have been reviewed from five pathologists.

**Figure 1:**
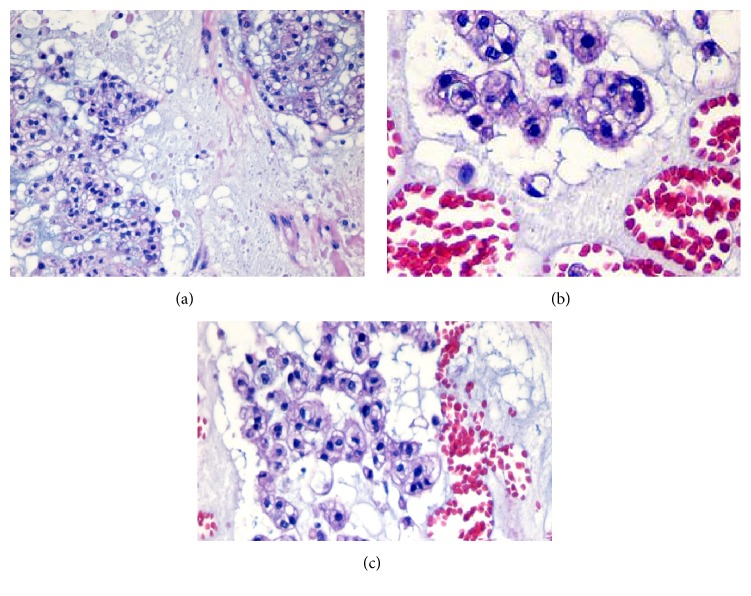
Neoplastic pleomorphic cells and physaliphorous cells, with vacuolated PAS positive cytoplasm and prominent vesicular nuclei with mild atypia, combined to small tumor cells with inconspicuous and no visible nuclei (haematoxylin and eosin, 20x and 40x).

**Figure 2:**
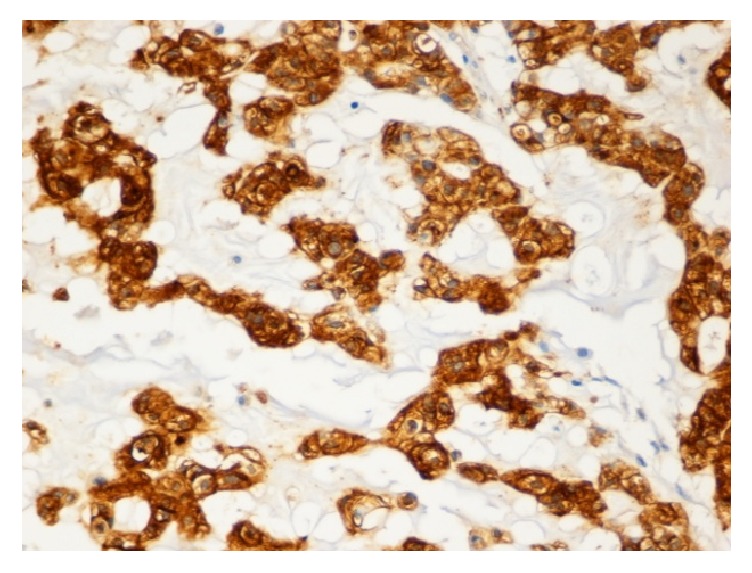
Positivity of the tumor cells for EMA (20x).

**Figure 3:**
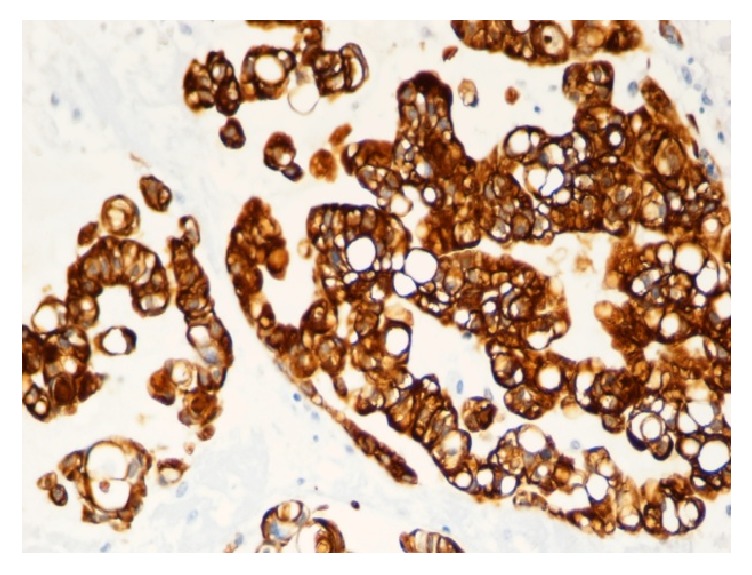
Positivity of the tumor cells for CKAE1/AE3 (20x).

**Figure 4:**
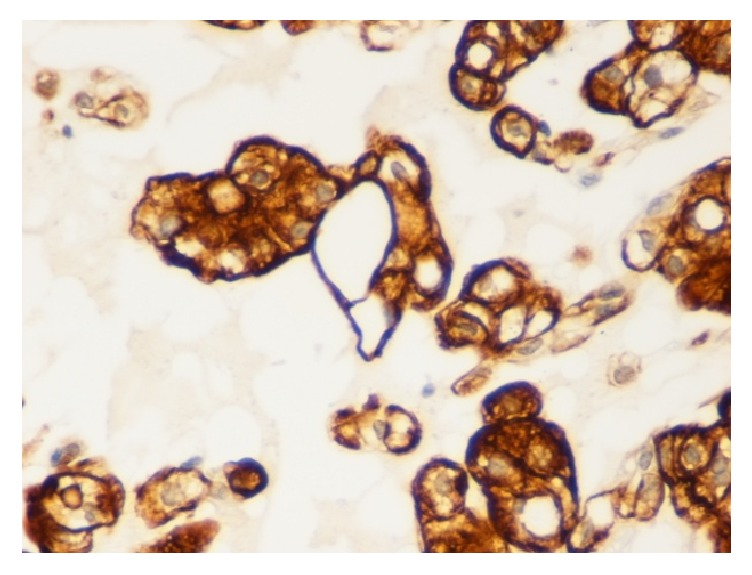
Positivity of the tumor cells for vimentin (40x).

**Figure 5:**
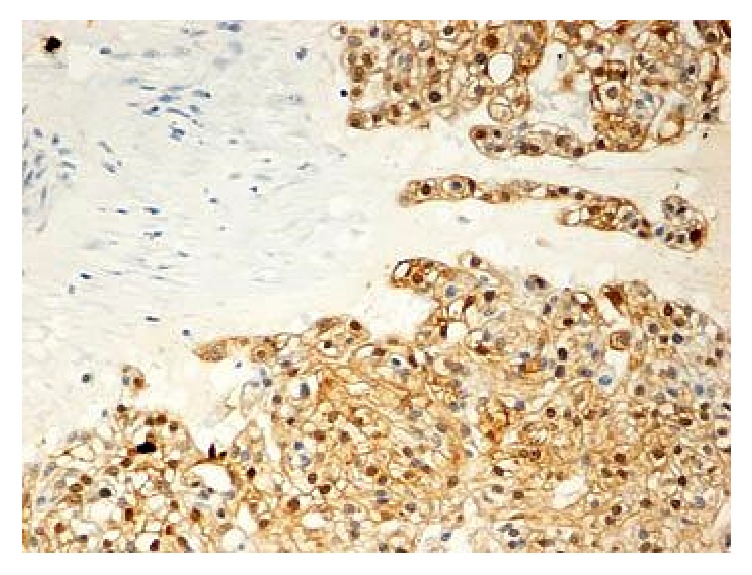
Positivity of the tumor cells for S100 (20x).

Histologically, the lesion was mainly composed of extremely large cells (known as* physaliphorous cells*), with vacuolated PAS positive cytoplasm and prominent vesicular nuclei with mild atypia, combined to small tumor cells with inconspicuous and no visible nuclei. Neoplastic cells showed consistent pleomorphism, shape, and cytoplasmic features [8]. These two populations seemed to be organized in cell cords and lobules separated by fibrous septae, within a mucoid intercellular matrix. Mitotic figures were scanty or absent. Neither foci of chondroid (cartilaginous) differentiation nor sarcomatous areas were observed as well (Figures 1(a)–1(c)). Tumor cells stained for S-100 protein (Figure 5), cytokeratin AE1/AE3 (Figure 3), EMA (Figure 2), and vimentin (Figure 4) whereas a negativity for CEA and smooth muscle actin was detected. Finally, a diagnosis of cutaneous metastasis of sacral chordoma was posed.

## 3. Discussion

Chordomas are rare tumors, accounting for 3% of all bone tumors. They usually arise from embryonic notochordal remnants along the length of the neuraxis at developmental active sites represented by the ends of the neuraxis and the vertebral bodies. The natural history of chordoma is characterized by repeated episodes of local recurrence and an often fatal outcome. This tumors shows a slow growth, with local destruction of the bone and extension to adjacent soft tissue. Recurrences may develop 10 years or longer after the initial therapy [9]. The frequency of metastasis was estimated in the literature with strongly divergent values ranging between 3% and 48%. The usual sites of metastases include lungs (52%), liver (23%), lymph nodes (20%), and bone (16%). Skin, subcutaneous tissue, and skeletal muscle are rare metastatic sites. The skin is usually involved for direct extension of the primary tumor. True distant hematogenous metastases to skin have been very rarely described. Occasionally they precede the diagnosis of the primary tumor [10]. Primary neoplasm and metastases show similar histological features.

By morphology, chordomas are classified into 3 subtypes:* conventional chordoma* which consists of eosinophilic pleomorphic cells embedded in a mucinous matrix, arranged in a diffuse or lobular pattern, or clustered in cords or nests;* chondroid chordoma*, predominantly observed in the sphenooccipital region, presenting foci of cartilaginous differentiation within areas of conventional chordoma;* dedifferentiated (sarcomatoid) chordoma*, which has a poor prognosis and shows areas of conventional chordoma associated with sarcomatous areas which commonly resemble malignant fibrous histiocytoma; the pathognomonic finding is represented by the so-called* physaliphorous* (Greek, bubbly) cells, which are characterized by voluminous, vacuolated, clear cytoplasm, with a peripheral nucleus, reflecting the notochordal cells in their late stage [4].

Several differential diagnoses should be excluded, such as myxoid chondrosarcoma, myxoid liposarcoma, and parachordoma [10], but the presence of* physaliphorous *cells and the peculiar immunohistochemical features, for example, positivity for cytokeratins, vimentin, epithelial membrane antigen (EMA), and S-100 protein, can be helpful for achieving the correct one. The immunoreactivity for cytokeratins and EMA can also be helpful in distinguishing the chondroid variant of chordoma from chondrosarcoma. Positivity for neuron specific enolase (NSE) has also been reported [11].

A genetic basis has been described for some chordomas which exhibit abnormal karyotypes like whole or partial losses of chromosomes 3, 4, 10 and 13, gains in chromosome 7, and rearrangements of chromosome 1p12.

Surgery represents the best treatment in order to remove the tumor and the infiltrated tissues around it but it depends on the site and its proximity to nervous components, vessels, and nerves [1, 12, 13]. Advancing in radiation therapies is permitting the somministration of higher doses of radiation without damaging to surrounding tissues. They include carbon ion beams therapy and intraoperative radiation which provide considerable outcome in terms of minimized toxicity [14]. Aggressive surgical excision followed by radiation therapy represents the best chance of long-term control [15]. Traditional chemotherapy has not been very effective due to its low efficacy [16]. No effective medical therapy is available [17]. Recent studies have focused on molecular targets expressed in the neoplastic cells of chordoma like platelet-derived growth factor receptor-alfa and beta (PDGFRA and PDGFRB). A clinical trial using imatinib mesylate, a tyrosine kinase inhibitor targeting PDGFRB, demonstrated a benefit in tumor response because of its antitumoral activity in patients with chordomas [18].

The evidence of AKT activation in a small number of chordoma patients prompted the oncologist to combine sirolimus, an mTOR inhibitor, to imatinib obtaining a reestablished tumor response [19]. Although chordomas show a benign appearance, they display a malignant behaviour, due to their capability of local invasion. The 5-year survival rate is 51% and the 10-year overall survival is estimated around 35%. Young age, complete resection, and radiation therapy in incompletely resected tumors represent favorable prognostic factors. Recent studies have focused on the presence of cell cycle alterations which led to an increase of MIB-1, nuclear pleomorphism, and p53 tumor suppressor gene overexpression in chordoma tumorigenesis [20]. p53 overexpression seems to be associated with an unfavorable outcome in patients with chordoma [20].

In up-to-date medical literature about 20 cases of cutaneous metastasis from sacrococcygeal chordoma were described [21]. The last one was illustrated in last year [22].

## 4. Conclusion

The interest in the present case lies in the fact that this is a true, very rare, cutaneous hematogenous metastasis arising in the absence of local recurrence, appearing after a long time interval from diagnosis of the primary tumor (10 years), characteristic of this type of malignancies.

Histological and immunohistochemical findings are close to those reported in literature. Cutaneous metastasis has been surgically removed and the lateral and deep resection margins showed no evidence of tumor infiltration. Up to now, any recurrence from the primary site has been detected. Although the occurrence of cutaneous metastases from chordoma is a rare event, an early and accurate clinical diagnosis is recommended.
